# Prognostic significance of stress hyperglycemia ratio in acute coronary syndrome patients with prior coronary artery bypass grafting

**DOI:** 10.3389/fendo.2025.1741291

**Published:** 2026-01-16

**Authors:** Xiaoteng Ma, Huijun Chu, Qiuxuan Li, Yuxiu Yang, Yujie Zhou, Zhijian Wang

**Affiliations:** 1 Department of Cardiology, Beijing Anzhen Hospital, Capital Medical University, Beijing, China; 2 Department of Anesthesia, Beijing Anzhen Hospital, Capital Medical University, Beijing, China

**Keywords:** acute coronary syndrome, coronary artery bypass grafting, major adverse cardiovascular and cerebrovascular events, percutaneous coronary intervention, stress hyperglycemia ratio

## Abstract

**Background:**

Patients with prior coronary artery bypass grafting (CABG) presenting with an acute coronary syndrome (ACS) constitute a subgroup at high cardiovascular risk and have a poor prognosis even after percutaneous coronary intervention (PCI). The stress hyperglycemia ratio (SHR) is a novel marker reflecting acute hyperglycemia adjusted for chronic glycemic status, but its prognostic value in this specific population remains unknown. This study aimed to investigate the association of SHR with long-term adverse cardiovascular outcomes in ACS patients with prior CABG.

**Methods:**

The SHR was calculated using the following formula: admission fasting blood glucose (AFBG)/[1.59 × glycosylated hemoglobin A1c (HbA1c) - 2.59]. The primary endpoint was the long-term incidence of major adverse cardiovascular and cerebrovascular events (MACCE), a composite of all-cause death, non-fatal stroke, non-fatal myocardial infarction, or unplanned revascularization.

**Results:**

A total of 1,208 ACS patients with prior CABG who underwent PCI were included in the final analysis. During a median follow-up of 1,291 days, 368 (30.5%) patients developed at least one primary endpoint event. Kaplan-Meier analysis revealed a graded, positive relationship between the SHR tertiles and the follow-up incidence of MACCE (log-rank *P* < 0.001). In multivariate Cox proportional hazards regression analysis adjusted for GRACE risk score and other confounders, compared with those in the lowest SHR tertile, patients in the middle and highest tertiles had a higher risk of MACCE (adjusted hazard ratio [HR]: 1.557, 95% confidence interval [CI] 1.166-2.079, *P* = 0.003, and 1.943, 95% CI 1.476-2.557, *P* < 0.001, respectively). Similar results were obtained when SHR was analyzed as a continuous variable (adjusted HR per unit increase 1.276, 95% CI 1.105-1.474, *P* = 0.001). The addition of SHR to the baseline reference prediction model including GRACE risk score improved model predictive performance markedly (C-statistic: increased from 0.559 to 0.626, *P* = 0.002; cNRI: 0.580, *P* = 0.016; IDI: 0.133, *P* = 0.010).

**Conclusions:**

In ACS patients with prior CABG undergoing PCI, an elevated SHR was a strong and independent predictor of long-term MACCE. This simple metric provides potent prognostic information, potentially enhancing risk stratification and guiding management in this high-risk patient population.

## Background

The management of patients with prior coronary artery bypass graft (CABG) surgery presenting with an acute coronary syndrome (ACS) who represent a particularly high-risk subgroup presents distinctive clinical challenges that go far beyond the scope of conventional coronary artery diseases because these patients were excluded from pivotal trials (1, 2). This specific patient population exhibits accelerated atherosclerosis progression in both native coronary arteries and bypass grafts, coupled with more complex lesion characteristics and frequent multivessel involvement (3–6). Current ACS guidelines recommend initial evaluation by cardiac catheterization in patients with prior CABG, followed by coronary revascularization if indicated (7, 8). For patients with previous CABG requiring repeat coronary revascularization, percutaneous coronary intervention (PCI) is the preferred option rather than repeat CABG (9). Despite technical advancements in interventional cardiology, when patients with prior CABG presenting with an ACS require PCI, they face substantially worse outcomes compared to those without prior CABG, including higher rates of periprocedural complications, recurrent ischemia, and long-term mortality (10, 11), highlighting the need for improved risk stratification tools.

Acute elevation of admission blood glucose is common in patients with ACS and is associated with adverse cardiovascular outcomes (12, 13). Hyperglycemia after ACS may be related to both background blood glucose and various stress mechanisms. Stress hyperglycemia refers to an acute increase in blood glucose levels adjusted for background glycemia during acute illness, regardless of whether diabetes is present or not (14, 15). The stress hyperglycemia ratio (SHR), calculated as admission glucose divided by estimated average glucose derived from glycosylated hemoglobin A1c (HbA1c), has emerged as a novel biomarker that can effectively reflect the state of stress hyperglycemia (16). This ratio provides a more accurate reflection of true acute metabolic stress than isolated glucose measurements, and has recently been recognized as a significant prognostic factor in patients with cardiovascular and cerebrovascular diseases (15, 17). The proposed mechanisms linking elevated SHR to adverse cardiovascular outcomes include exacerbated endothelial dysfunction, enhanced platelet reactivity, increased oxidative stress, and profound inflammatory activation (18). These pathological processes may be particularly detrimental in ACS patients with prior CABG, who often present with pre-existing endothelial damage and compromised vascular integrity (19, 20).

The pathophysiology of post-CABG patients, characterized by excessive inflammatory response, microvascular dysfunction, and accelerated graft atherosclerosis (5, 6, 21–24), may create a vulnerable substrate where stress hyperglycemia following ACS exerts particularly harmful effects. Of note, PCI may complicate the pathophysiology of ACS patients with prior CABG (5). Numerous studies have confirmed the prognostic value of SHR in patients with ACS undergoing PCI or those undergoing CABG (17, 25–27). However, the specific prognostic significance of SHR in ACS patients with prior CABG who underwent PCI remains unexplored, representing a critical gap in current cardiovascular risk assessment methodologies.

This study aims to investigate the association of SHR with adverse cardiovascular outcomes in ACS patients with prior CABG undergoing PCI, with the hypothesis that elevated SHR independently predicts increased risk of long-term major adverse cardiovascular and cerebrovascular events (MACCE) in this patient population.

## Methods

### Study design and population

The present study is a retrospective analysis derived from a single-center retrospective observational cohort that originally aimed to investigate the association between the PCI target-vessel (native coronary arteries vs. bypass grafts) and adverse cardiovascular events (NCT05368597). We screened 1,435 consecutive patients with a history of prior CABG who were hospitalized for an ACS and underwent PCI between September 2008 and October 2021. The diagnosis of ACS, including unstable angina (UA), non-ST-segment elevation myocardial infarction (NSTEMI), and ST-segment elevation myocardial infarction (STEMI), was established according to contemporary clinical guidelines (28, 29). For the purpose of this study, the following patients were excluded: those who died prior to discharge, those undergoing hybrid coronary revascularization, those with left ventricular ejection fraction (LVEF) < 30%, renal dysfunction with an estimated glomerular filtration rate (eGFR) < 30 mL/min/1.73m^2^ or chronic dialysis, those who were currently using glucocorticoids for connective tissue disease, those who had active infections on admission, and those lacking relevant baseline data for analysis. A final cohort of 1,208 patients was included in the analysis. The study protocol was approved by the local Institutional Review Board of Beijing Anzhen Hospital, Capital Medical University (No. 2025320x). The study was conducted in accordance with the ethical principles of the Declaration of Helsinki. Given the retrospective nature of the study, the requirement for informed consent was waived.

### Data collection and definitions

Comprehensive baseline data were meticulously extracted from the hospital’s electronic medical records system. Collected information included:

Demographics and Vital Signs: Age, sex, body mass index (BMI), systolic blood pressure (SBP), and heart rate at admission.

Medical History: Hypertension, diabetes, heart failure, renal dysfunction, atrial fibrillation, previous myocardial infarction (MI), past PCI, previous stroke, peripheral artery disease (PAD), chronic lung disease, and family history of coronary artery disease (CAD). Heart failure was defined as signs or symptoms of heart failure, treatment for heart failure, or LVEF < 40%. Renal dysfunction was defined as eGFR < 60ml/min/1.73m^2^, calculated by the Chronic Kidney Disease Epidemiology Collaboration (CKD-EPI) equation. Previous stroke was defined as previous cerebral infarction or hemorrhage.

Clinical Presentation: Type of ACS (UA, NSTEMI, or STEMI). The GRACE (Global Registry of Acute Coronary Events) risk score (based on the GRACE 6-month post-discharge prediction model) was calculated for each patient to standardize the assessment of baseline ischemic risk.

Laboratory Parameters: All laboratory measurements were performed at the central laboratory of Beijing Anzhen Hospital on the first fasting blood samples drawn after admission, following a mandated fasting period of at least 8 hours. These included serum creatinine (SCr), lipid profile (total cholesterol [TC], low density lipoprotein cholesterol [LDL-C], high density lipoprotein cholesterol [HDL-C], triglycerides), high-sensitivity C-reactive protein (hs-CRP), fasting blood glucose (FBG), and HbA1c.

Procedural Details: Time from prior CABG to the index PCI, vascular access site (radial/brachial vs. femoral), and target vessels for PCI (native coronary arteries and/or bypass grafts).

Medication Use: Use of hypoglycemic agents (insulin and oral drugs) before admission and guideline-directed medical therapies at discharge, including antiplatelets, statins, β-blockers, and angiotensin-converting enzyme inhibitors/angiotensin receptor blockers/angiotensin receptor-neprilysin inhibitors (ACEI/ARB/ARNIs).

### Calculation of SHR

The key exposure variable, SHR, was calculated using the following formula: admission FBG (AFBG)/[1.59 × HbA1c - 2.59]. Based on the SHR values, the entire study population was stratified into three groups: the lowest tertile (SHR ≤ 0.7700), the middle tertile (0.7700 < SHR ≤ 0.8899), and the highest tertile (SHR > 0.8899).

### Study outcomes

Information on adverse cardiovascular outcomes was obtained by trained personnel with no knowledge of baseline characteristics through telephone contact with the patients or their family members and was determined by careful review of the corresponding medical records. The primary endpoint was the occurrence of MACCE, a composite of all-cause death, non-fatal stroke, non-fatal MI, or unplanned revascularization during the follow-up period. Secondary endpoints included the individual components of the primary endpoint and a key secondary composite endpoint of all-cause death, non-fatal stroke, or non-fatal MI. The final follow-up was conducted in April 2022.

### Statistical analysis

Continuous variables were presented as mean ± standard deviation (SD) for normally distributed data or median (interquartile range, IQR) for skewed data. Comparisons across three groups were made using one-way ANOVA or the Kruskal-Wallis test, and between two groups were made using t-test or Mann-Whitney U test. Categorical variables were expressed as numbers (percentages) and were compared using the chi-square test or Fisher’s exact test.

The SHR was primarily analyzed as a categorical variable (i.e., the SHR tertiles) and a continuous variable for its association with MACCE. Additionally, receiver operating characteristic (ROC) curve analysis and Youden’s index (sensitivity + specificity − 1) were used to determine the optimal cutoff value of SHR as a continuous variable for predicting the occurrence of MACCE. The cumulative incidence of clinical endpoints over time was visualized using Kaplan-Meier survival curves, and differences among the SHR tertiles were assessed with the log-rank test. To evaluate the independent prognostic value of SHR, both univariate and multivariate Cox proportional hazards regression models were used to estimate the hazard ratios (HRs) with corresponding 95% confidence intervals (CIs) for time to first occurrence of the primary endpoint. Two sets of multivariate models were constructed: one included the GRACE risk score, and a sensitivity analysis excluded it and included its components. Variables with a univariate significance level of < 0.20 were included in the multivariate Cox proportional hazards regression model, while those that might cause internal correlations were excluded. The C-statistic, continuous net reclassification improvement (cNRI), and integrated discrimination improvement (IDI) were calculated to assess the incremental predictive value of adding SHR to the baseline reference prediction models. The model’s discriminatory performance is assessed using the C-statistic, which is equivalent to the area under the receiver operating characteristic curve, with values closer to 1.0 indicating better discrimination. The cNRI quantifies the correct reclassification of patient risk (both with and without events) when the new biomarker is added. The IDI summarizes the overall improvement in predicted probabilities for events and non-events. A positive cNRI and IDI with a P-value < 0.05 indicates a significant improvement in model performance. Furthermore, *post-hoc* subgroup analyses were performed to assess the consistency of the association between SHR (as a continuous variable) and MACCE across various patient subgroups. The interaction between the subgroup variable and SHR was tested. A two-sided *P*-value < 0.05 was considered statistically significant. All statistical analyses were conducted using SPSS version 24.0 (IBM Corp., Armonk, New York, US) and R software version 4.1.0 (R Foundation for Statistical Computing, Beijing, China). The calculation of C-statistics and the comparison between two groups were performed using the “survival”, “prodlim” and “survcomp” packages in R; while the calculations of cNRI and IDI were performed using the “survival”, “survC1” and “survIDINRI” packages. The Kaplan-Meier curves were plotted using GraphPad Prism version 7.0 (GraphPad Software Inc., San Diego, California, US).

## Results

### Baseline characteristics stratified by the SHR tertiles

Of the initial 1,435 patients, only 2 died during hospitalization (the cause of death was related to PCI procedure), and these 2 patients were excluded from this analysis. The baseline characteristics of the 1,208 patients included in the study, categorized into the lowest (n = 402), middle (n = 404), and highest (n = 402) SHR tertiles, are summarized in **Table 1**. The three groups were well-balanced in terms of age and most traditional cardiovascular risk factors, such as hypertension and smoking status. However, significant differences emerged. The prevalence of diabetes was higher in the lowest and highest SHR tertiles (62.7% and 61.4%, respectively), compared to the middle tertile (47.0%, *P* < 0.001). Patients in the highest SHR tertile presented with a higher heart rate at admission (69 ± 11 bpm vs. 67 ± 9 and 66 ± 10 bpm in the other groups, *P* = 0.003) and a more pronounced inflammatory and metabolic profile, characterized by significantly higher levels of hs-CRP, triglycerides, and AFBG (all *P* < 0.001). Interestingly, HbA1c levels were lowest in the middle SHR tertile. The highest SHR tertile group underwent PCI in graft vessels, particularly saphenous vein grafts (SVGs), more frequently than the other groups (PCI in only graft vessels: 14.4% vs. 7.7% and 8.4%; SVG intervention: 19.9% vs. 11.4% and 13.1%; *P* < 0.01 for both). Consequently, the use of hypoglycemic agents, both before admission and at discharge (including insulin and oral drugs), was significantly more common in the highest SHR tertile (all *P* < 0.001). Discharge medications, including antiplatelets, statins, and β-blockers, were otherwise similar across all groups.

**Table 1:** Baseline characteristics of the study population according to the SHR tertiles.

Variable	All patients n = 1,208	Lowest tertile n = 402	Middle tertile n = 404	Highest tertile n = 402	*P* value
SHR, median (IQR)	0.8188 (0.7389-0.9455)	0.7042 (0.6428-0.7389)	0.8188 (0.7932-0.8520)	1.0359 (0.9455-1.2358)	<0.001
Age (years), mean ± SD	64 ± 8	64 ± 8	64 ± 8	64 ± 8	0.757
Male sex, n (%)	914 (75.7)	291 (72.4)	325 (80.4)	298 (74.1)	0.020
BMI (kg/m^2^), mean ± SD	26.2 ± 3.2	26.1 ± 3.1	26.3 ± 3.2	26.3 ± 3.2	0.560
SBP at admission (mmHg), mean ± SD	130 ± 17	130 ± 17	130 ± 18	130 ± 17	0.946
Heart rate at admission (bpm), mean ± SD	67 ± 10	67 ± 9	66 ± 10	69 ± 11	0.003
Family history of CAD, n (%)	120 (9.9)	44 (10.9)	29 (7.2)	47 (11.7)	0.071
Current smoking, n (%)	290 (24.0)	100 (24.9)	94 (23.3)	96 (23.9)	0.865
Hypertension, n (%)	930 (77.0)	309 (76.9)	307 (76.0)	314 (78.1)	0.773
Diabetes, n (%)	689 (57.0)	252 (62.7)	190 (47.0)	247 (61.4)	<0.001
HF, n (%)	108 (8.9)	35 (8.7)	33 (8.2)	40 (10.0)	0.662
Renal dysfunction, n (%)	94 (7.8)	29 (7.2)	24 (5.9)	41 (10.2)	0.068
AF, n (%)	45 (3.7)	12 (3.0)	20 (4.9)	13 (3.2)	0.272
Previous MI, n (%)	594 (49.2)	195 (48.5)	192 (47.5)	207 (51.5)	0.503
Past PCI, n (%)	345 (28.6)	108 (26.9)	121 (30.0)	116 (28.9)	0.617
Previous stroke, n (%)	148 (12.3)	44 (10.9)	52 (12.9)	52 (12.9)	0.620
PAD, n (%)	119 (9.9)	42 (10.4)	43 (10.6)	34 (8.5)	0.515
Chronic lung disease, n (%)	56 (4.6)	15 (3.7)	23 (5.7)	18 (4.5)	0.409
Clinical presentation					0.130
UA, n (%)	1,019 (84.4)	350 (87.1)	343 (84.9)	326 (81.1)	
NSTEMI, n (%)	152 (12.6)	44 (10.9)	50 (12.4)	58 (14.4)	
STEMI, n (%)	37 (3.1)	8 (2.0)	11 (2.7)	18 (4.5)	
GRACE risk score, mean ± SD	89 ± 21	89 ± 20	89 ± 21	91 ± 23	0.214
Laboratory measurements (fasting state)					
SCr (umol/L), mean ± SD	77.1 ± 18.8	75.6 ± 18.1	77.7 ± 19.3	78.0 ± 18.9	0.155
TC (mmol/L), mean ± SD	3.99 ± 1.07	3.93 ± 1.16	3.98 ± 1.01	4.06 ± 1.05	0.207
LDL-C (mmol/L), mean ± SD	2.34 ± 0.87	2.29 ± 0.89	2.35 ± 0.91	2.36 ± 0.82	0.499
HDL-C (mmol/L), mean ± SD	0.99 ± 0.23	0.99 ± 0.22	1.01 ± 0.23	0.98 ± 0.24	0.206
Triglycerides (mmol/L), median (IQR)	1.56 (1.14-2.12)	1.47 (1.10-2.02)	1.48 (1.09-2.03)	1.75 (1.27-2.26)	<0.001
Hs-CRP (mg/L), median (IQR)	1.38 (0.69-3.39)	1.23 (0.60-3.26)	1.33 (0.64-3.47)	1.62 (0.82-3.36)	0.012
FPG (mmol/L), median (IQR)	6.26 (5.37-8.01)	5.43 (4.90-6.30)	5.97 (5.36-7.04)	8.54 (6.79-12.50)	<0.001
HbA1c (%), median (IQR)	6.4 (5.9-7.4)	6.6 (6.1-7.5)	6.2 (5.8-7.0)	6.5 (5.8-7.7)	<0.001
Years from CABG, median (IQR)	6.0 (3.0-10.0)	6.0 (3.0-10.0)	6.0 (2.0-10.0)	6.0 (3.0-10.0)	0.420
The index PCI as the first PCI after CABG, n (%)	1106 (91.6)	371 (92.3)	367 (90.8)	368 (91.5)	0.761
Percutaneous entry					0.671
Radial/brachial only, n (%)	608 (50.3)	213 (53.0)	198 (49.0)	197 (49.0)	
Femoral only, n (%)	554 (45.9)	177 (44.0)	189 (46.8)	188 (46.8)	
Radial/brachial + Femoral, n (%)	46 (3.8)	12 (3.0)	17 (4.2)	17 (4.2)	
Procedure results					
PCI in native and/or graft vessels					0.005
PCI in only native vessels, n (%)	1025 (84.9)	355 (88.3)	351 (86.9)	319 (79.4)	
PCI in only graft vessels, n (%)	123 (10.2)	31 (7.7)	34 (8.4)	58 (14.4)	
PCI in both native and graft vessels, n (%)	60 (5.0)	16 (4.0)	19 (4.7)	25 (6.2)	
Native vessel intervened, n (%)					
LM	144 (11.9)	47 (11.7)	54 (13.4)	43 (10.7)	0.497
LAD	295 (24.4)	99 (24.6)	98 (24.3)	98 (24.4)	0.992
LCX	420 (34.8)	128 (31.8)	154 (38.1)	138 (34.3)	0.169
RCA	577 (47.8)	204 (50.7)	190 (47.0)	183 (45.5)	0.312
Graft vessel intervened, n (%)					
LIMA	4 (0.3)	1 (0.2)	0 (0)	3 (0.7)	0.135
SVG	179 (14.8)	46 (11.4)	53 (13.1)	80 (19.9)	0.002
Use of hypoglycemic agents before admission, n (%)	526 (43.5)	180 (44.8)	130 (32.2)	216 (53.7)	<0.001
Use of medications at discharge					
Aspirin, n (%)	1198 (99.2)	397 (98.8)	403 (99.8)	398 (99.0)	0.218
P2Y12 inhibitors, n (%)	1198 (99.2)	400 (99.5)	398 (98.5)	400 (99.5)	0.368
Oral anticoagulants, n (%)	37 (3.1)	13 (3.2)	14 (3.5)	10 (2.5)	0.702
Statins, n (%)	1197 (99.1)	398 (99.0)	402 (99.5)	397 (98.8)	0.488
β-blockers, n (%)	1021 (84.5)	339 (84.3)	341 (84.4)	341 (84.8)	0.978
ACEI/ARB/ARNIs, n (%)	638 (52.8)	207 (51.5)	206 (51.0)	225 (56.0)	0.297
Insulin, n (%)	257 (21.3)	102 (25.4)	55 (13.6)	100 (24.9)	<0.001
Oral hypoglycemic agents, n (%)	413 (34.2)	135 (33.6)	106 (26.2)	172 (42.8)	<0.001

ACEI/ARB/ARNIs, angiotensin converting enzyme inhibitors/angiotensin receptor blockers/angiotensin receptor-neprilysin inhibitors; AF, atrial fibrillation/flutter; BMI, body mass index; CABG, coronary artery bypass grafting; CAD, coronary artery disease; FBG, fasting blood glucose; HbA1c, glycated hemoglobin A1c; HDL-C, high-density lipoprotein-cholesterol; HF, heart failure; HsCRP, high-sensitivity C-reactive protein; LAD, left anterior descending artery; LCX, left circumflex artery; LDL-C, low-density lipoprotein-cholesterol; LIMA, left internal mammary artery; LM, left main coronary artery; LVEF, left ventricular ejection fraction; MI, myocardial infarction; NSTEMI, non ST-segment elevation myocardial infarction; PAD, peripheral artery disease; PCI, percutaneous coronary intervention; RCA, right coronary artery; SBP, systolic blood pressure; SCr, serum creatinine; SHR, stress hyperglycemia ratio; STEMI, ST-segment elevation myocardial infarction; SVG, saphenous vein graft; TC, total cholesterol; TIA, transient ischemic attack; UA, unstable angina.

### Baseline characteristics stratified by MACCE

Patients were followed up for a median of 1,291 days (750–2008 days). During the period 368 patients (30.5%) experienced MACCE. The baseline characteristics of the study population stratified by MACCE are shown in **Table 2**. Compared to patients without MACCE, those with MACCE had a significantly higher median SHR (0.8658 vs. 0.8005, *P* < 0.001). The distribution of MACCE across the SHR tertiles was striking: 20.7% in the lowest, 33.4% in the middle, and 45.9% in the highest tertile. Patients who developed MACCE had a less favorable risk profile at baseline, including higher BMI, SBP, and heart rate at admission. They also had a greater burden of comorbidities, such as hypertension, renal dysfunction, and past PCI. Patients with MACCE had a more atherogenic lipid profile (higher LDL-C and triglycerides, lower HDL-C), and higher levels of hs-CRP and AFBG (all *P* < 0.05).

**Table 2:** Baseline characteristics of the study population grouped by MACCE.

Variable	Patients without MACCE n = 840	Patients with MACCE n = 368	*P* value
SHR, median (IQR)	0.8005 (0.7253-0.9113)	0.8658 (0.7842-1.0221)	<0.001
Lowest tertile, n (%)	326 (38.8)	76 (20.7)	
Middle tertile, n (%)	281 (33.5)	123 (33.4)	
Highest tertile, n (%)	233 (27.7)	169 (45.9)	
Age (years), mean ± SD	64 ± 8	65 ± 8	0.437
Male sex, n (%)	641 (76.3)	273 (74.2)	0.428
BMI (kg/m^2^), mean ± SD	26.1 ± 3.1	26.5 ± 3.4	0.021
SBP at admission (mmHg), mean ± SD	129 ± 17	132 ± 18	0.033
Heart rate at admission (bpm), mean ± SD	67 ± 10	68 ± 10	0.011
Family history of CAD, n (%)	73 (8.7)	47 (12.8)	0.029
Current smoking, n (%)	193 (23.0)	97 (26.4)	0.205
Hypertension, n (%)	632 (75.2)	298 (81.0)	0.029
Diabetes, n (%)	470 (56.0)	219 (59.5)	0.250
HF, n (%)	76 (9.0)	32 (8.7)	0.844
Renal dysfunction, n (%)	52 (6.2)	42 (11.4)	0.002
AF, n (%)	31 (3.7)	14 (3.8)	0.923
Previous MI, n (%)	405 (48.2)	189 (51.4)	0.314
Past PCI, n (%)	224 (26.7)	121 (32.9)	0.028
Previous stroke, n (%)	98 (11.7)	50 (13.6)	0.349
PAD, n (%)	79 (9.4)	40 (10.9)	0.432
Chronic lung disease, n (%)	43 (5.1)	13 (3.5)	0.227
Clinical presentation			0.391
UA, n (%)	713 (84.9)	306 (83.2)	
NSTEMI, n (%)	105 (12.5)	47 (12.8)	
STEMI, n (%)	22 (2.6)	15 (4.1)	
GRACE risk score, median (IQR)	87 (75-103)	89 (75-103)	0.173
Laboratory measurements (fasting state)			
SCr (umol/L), mean ± SD	76.2 ± 17.0	79.3 ± 22.3	0.017
TC (mmol/L), mean ± SD	3.92 ± 1.04	4.16 ± 1.13	0.001
LDL-C (mmol/L), mean ± SD	2.27 ± 0.84	2.49 ± 0.93	<0.001
HDL-C (mmol/L), mean ± SD	1.01 ± 0.23	0.95 ± 0.23	<0.001
Triglycerides (mmol/L), median (IQR)	1.45 (1.07-2.02)	1.80 (1.36-2.30)	<0.001
HsCRP (mg/L), median (IQR)	1.10 (0.50-2.86)	2.13 (1.18-4.54)	<0.001
FBG (mmol/L), median (IQR)	5.96 (5.21-7.63)	6.86 (5.88-9.00)	<0.001
HbA1c (%), median (IQR)	6.4 (5.8-7.3)	6.5 (5.9-7.5)	0.025
Years from CABG, median (IQR)	6.0 (2.5-10.0)	6.0 (3.0-10.0)	0.268
The index PCI as the first PCI after CABG, n (%)	779 (92.7)	327 (88.9)	0.026
Percutaneous entry			0.098
Radial/brachial only, n (%)	440 (52.4)	168 (45.7)	
Femoral only, n (%)	369 (43.9)	185 (50.3)	
Radial/brachial + Femoral, n (%)	31 (3.7)	15 (4.1)	
Procedure results			
PCI in native and/or graft vessels			0.026
PCI in only native vessels, n (%)	722 (86.0)	303 (82.3)	
PCI in only graft vessels, n (%)	73 (8.7)	50 (13.6)	
PCI in both native and graft vessels, n (%)	45 (5.4)	15 (4.1)	
Native vessel intervened, n (%)			
LM	109 (13.0)	35 (9.5)	0.087
LAD	211 (25.1)	84 (22.8)	0.393
LCX	283 (33.7)	137 (37.2)	0.235
RCA	408 (48.6)	169 (45.9)	0.397
Graft vessel intervened, n (%)			
LIMA	3 (0.4)	1 (0.3)	1.000
SVG	115 (13.7)	64 (17.4)	0.096
Use of hypoglycemic agents before admission, n (%)	357 (42.5)	169 (45.9)	0.269
Use of medications at discharge			
Aspirin, n (%)	833 (99.2)	365 (99.2)	1.000
P2Y12 inhibitors, n (%)	833 (99.2)	365 (99.2)	1.000
Oral anticoagulants, n (%)	30 (3.6)	7 (1.9)	0.121
Statins, n (%)	832 (99.0)	365 (99.2)	1.000
β-blockers, n (%)	713 (84.9)	308 (83.7)	0.600
ACEI/ARB/ARNIs, n (%)	428 (51.0)	210 (57.1)	0.050
Insulin, n (%)	177 (21.1)	80 (21.7)	0.794
Oral hypoglycemic agents, n (%)	281 (33.5)	132 (35.9)	0.415

MACCE indicates major adverse cardiovascular and cerebrovascular events. Other abbreviations as in **Table 1**.

### Clinical outcomes and Kaplan-Meier analysis

**Figure 1:**
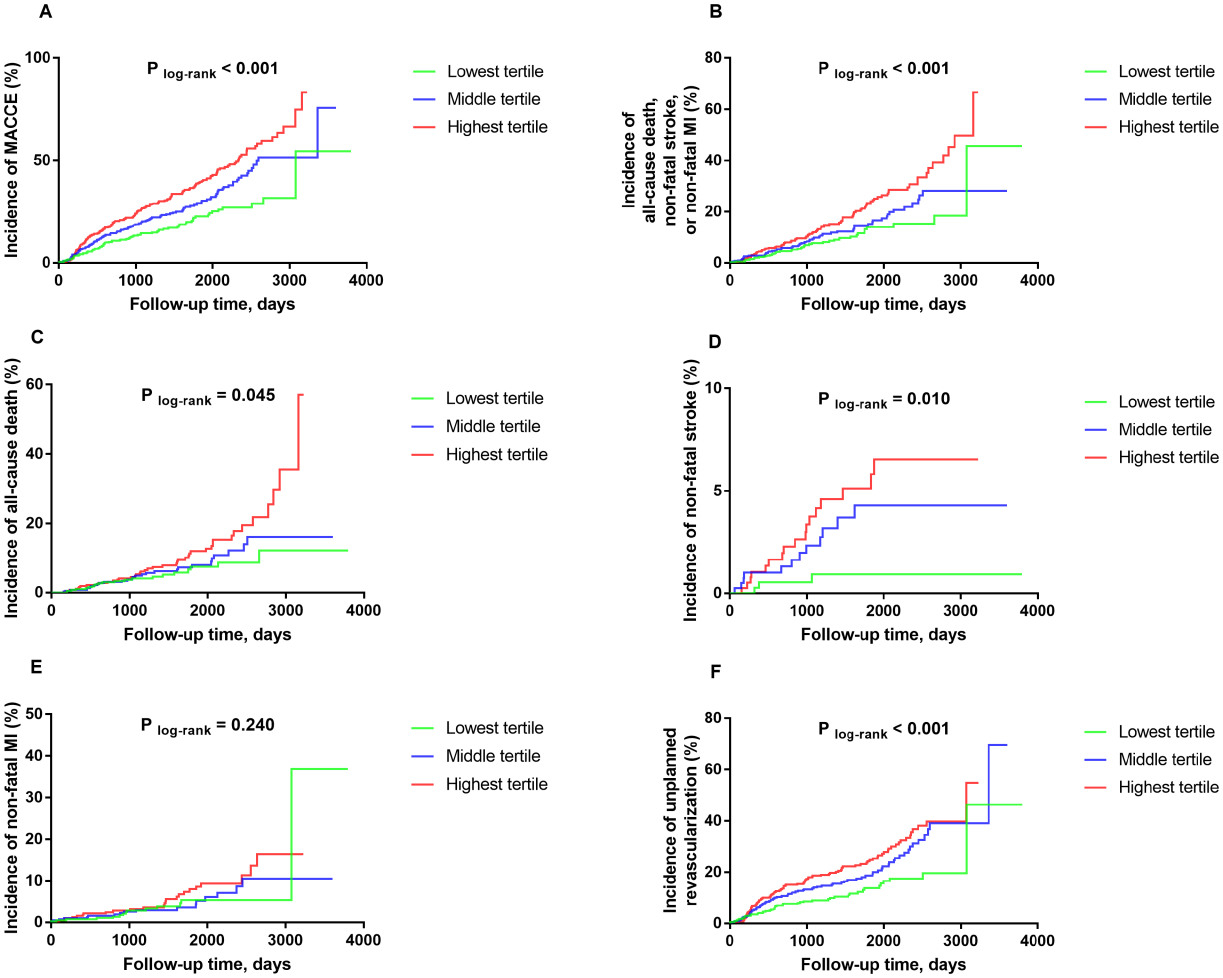
Kaplan-Meier curves for the incidences of MACCE **(A)**, the key secondary endpoint **(B)**, all-cause death **(C)**, non-fatal stroke **(D)**, non-fatal MI **(E)**, and unplanned revascularization **(F)** among the three study groups based on the SHR tertiles MACCE was defined as the composite of all-cause death, non-fatal stroke, non-fatal MI, and unplanned revascularization. The key secondary endpoint was defined as the composite of all-cause death, non-fatal stroke, and non-fatal MI. MACCE, major adverse cardiovascular and cerebrovascular events; MI, myocardial infarction.

The Kaplan-Meier curves vividly illustrated a graded, positive relationship between the SHR tertiles and the follow-up incidence of MACCE over the long-term follow-up. The cumulative incidence of MACCE was significantly higher with each increasing SHR tertile (log-rank *P* < 0.001; **Figure 1A**). This pattern was consistent for the key secondary endpoint (log-rank *P* < 0.001; **Figure 1B**). The difference in the incidence of MACCE was mainly driven by significant increases in all-cause death (log-rank *P* = 0.045; **Figure 1C**), non-fatal stroke (log-rank *P* = 0.010; **Figure 1D**), and unplanned revascularization (log-rank *P* < 0.001; **Figure 1F**). However, the incidence of non-fatal MI was not significantly different among the SHR tertiles (log-rank *P* = 0.240; **Figure 1E**).

### Cox proportional hazards regression analysis

In univariate analysis (**Table 3**), both the middle and highest SHR tertiles were strongly associated with an increased risk of MACCE compared to the lowest tertile (HR 1.557, 95% CI 1.169-2.072, *P* = 0.002; HR 2.145, 95% CI 1.636-2.812, *P* < 0.001, respectively). When analyzed as a continuous variable, each unit increase in SHR was associated with a 24% increased risk of MACCE (HR 1.239, 95% CI 1.103-1.392, *P* < 0.001; **Supplementary Table S1**).

**Table 3:** Univariate and multivariate Cox proportional hazards models including GRACE risk score for predicting MACCE according to the SHR tertiles.

	Univariate analysis		Multivariate analysis	
Variables	HR (95% CI)	P value	HR (95% CI)	P value
SHR		<0.001		<0.001
Lowest tertile	ref		ref	ref
Middle tertile	1.557 (1.169-2.072)	0.002	1.557 (1.166-2.079)	0.003
Highest tertile	2.145 (1.636-2.812)	<0.001	1.943 (1.476-2.557)	<0.001
GRACE risk score	1.006 (1.002-1.011)	0.009	1.004 (0.999-1.009)	0.139
BMI	1.033 (1.000-1.068)	0.048	1.022 (0.988-1.057)	0.209
Hypertension	1.302 (1.003-1.689)	0.047	1.166 (0.892-1.524)	0.261
Diabetes	1.171(0.951-1.443)	0.137	1.046 (0.842-1.299)	0.683
Renal dysfunction	1.522 (1.103-2.101)	0.011	1.294 (0.914-1.832)	0.146
Past PCI	1.312 (1.055-1.631)	0.014	1.238 (0.958-1.600)	0.103
Previous stroke	1.322 (0.980-1.782)	0.067	1.206 (0.888-1.639)	0.231
Chronic lung disease	0.654 (0.376-1.140)	0.134	0.630 (0.360-1.104)	0.106
LDL-C	1.208 (1.090-1.338)	<0.001	1.210 (1.084-1.350)	<0.001
HDL-C	0.526 (0.328-0.845)	0.008	0.650 (0.400-1.084)	0.100
Triglycerides	1.066 (1.013-1.121)	0.014	1.033 (0.965-1.105)	0.350
Hs-CRP	1.022 (1.008-1.037)	0.002	1.013 (0.998-1.028)	0.096
Years from CABG	1.033 (1.010-1.057)	0.004	1.018 (0.993-1.044)	0.167
The index PCI as the first PCI after CABG	0.714 (0.516-0.989)	0.043	0.901 (0.602-1.349)	0.613
PCI in native and/or graft vessels		0.038		0.332
PCI in only native vessels	ref		ref	
PCI in only graft vessels	1.434 (1.063-1.935)	0.018	1.708 (0.236-12.371)	0.596
PCI in both native and graft vessels	0.810 (0.482-1.362)	0.427	1.116 (0.145-8.574)	0.916
Native vessel intervened: LM	0.685 (0.483-0.971)	0.034	0.756 (0.530-1.079)	0.124
Graft vessel intervened: SVG	1.216 (0.928-1.592)	0.156	0.660 (0.090-4.829)	0.682

HR indicates hazard ratio; 95% CI, 95% confidence interval. Other abbreviations as in **Tables 1**, **2**.

The prognostic significance of a high SHR remained robust after extensive multivariate adjustment. In the primary model adjusting for the GRACE risk score and other confounders (**Table 3**), the middle and highest SHR tertiles persisted as powerful, independent predictors of MACCE (adjusted HR: 1.557, 95% CI 1.166-2.079, *P* = 0.003, and 1.943, 95% CI 1.476-2.557, *P* < 0.001, respectively). Similarly, SHR as a continuous variable retained its independent association with MACCE risk (adjusted HR: 1.276, 95% CI 1.105-1.474, *P* = 0.001; **Supplementary Table S1**). According to the ROC curve analysis and Youden’s index, the optimal cut-off value of SHR for predicting MACCE was 0.7889. Compared with those with SHR < 0.7889, patients with SHR ≥ 0.7889 were at higher risk of MACCE (adjusted HR: 1.694; 95% CI: 1.340-2.141; P < 0.001).

The consistency of the above findings was confirmed in sensitivity analyses that the COX models excluded the GRACE risk score and included its components, where the middle and highest SHR tertiles remained significantly and independently associated with MACCE (adjusted HR: 1.549, 95% CI 1.160-2.068, *P* = 0.003, and 1.909, 95% CI 1.448-2.517, *P* < 0.001; **Supplementary Table S2**). Similarly, SHR as a continuous variable retained its independent association with MACCE risk (adjusted HR: 1.276, 95% CI 1.104-1.474, *P* = 0.001; **Supplementary Table S3**).

### C-statistic, NRI, and IDI

The addition of SHR to the baseline reference prediction model including GRACE risk score improved model predictive performance markedly (C-statistic: increased from 0.559 [95% CI 0.567-0.631] to 0.626 [95% CI 0.597-0.656], *P* = 0.002; cNRI: 0.580 [95% CI 0.244-1.177], *P* = 0.016; IDI: 0.133 [95% CI 0.044-0.229], *P* = 0.010). Similarly, the addition of SHR had an incremental effect on the predictive ability of the baseline reference prediction model excluding GRACE risk score and including its components for MACCE (C-statistic: increased from 0.615 [95% CI 0.584-0.647] to 0.639 [95% CI 0.610-0.668], *P* = 0.003; cNRI: 0.556 [95% CI 0.242-1.180], *P* = 0.014; IDI: 0.118 [95% CI 0.043-0.209], *P* = 0.004). Compared with the baseline GRACE risk score, the addition of SHR had a significant increase in C-statistic from 0.540 (95% CI 0.506-0.574) to 0.590 (95% CI 0.560-0.620) (*P* = 0.003), and significant improvement in reclassification as assessed by the cNRI (0.654, 95% CI 0.215-1.048, *P* = 0.012) and IDI (0.158, 95% CI 0.054-0.270, *P* = 0.004).

The formula of SHR included AFBG and HbA1c. We compared the predictive abilities of SHR, AFBG and HbA1c for MACCE. The C-statistics of SHR, AFBG and HbA1c were 0.613 (0.569-0.658), 0.608 (0.578-0.639), and 0.550 (0.517-0.583), respectively. Based on the pairwise comparison of the C-statistics, SHR performed the best.

### Subgroup analysis

**Figure 2:**
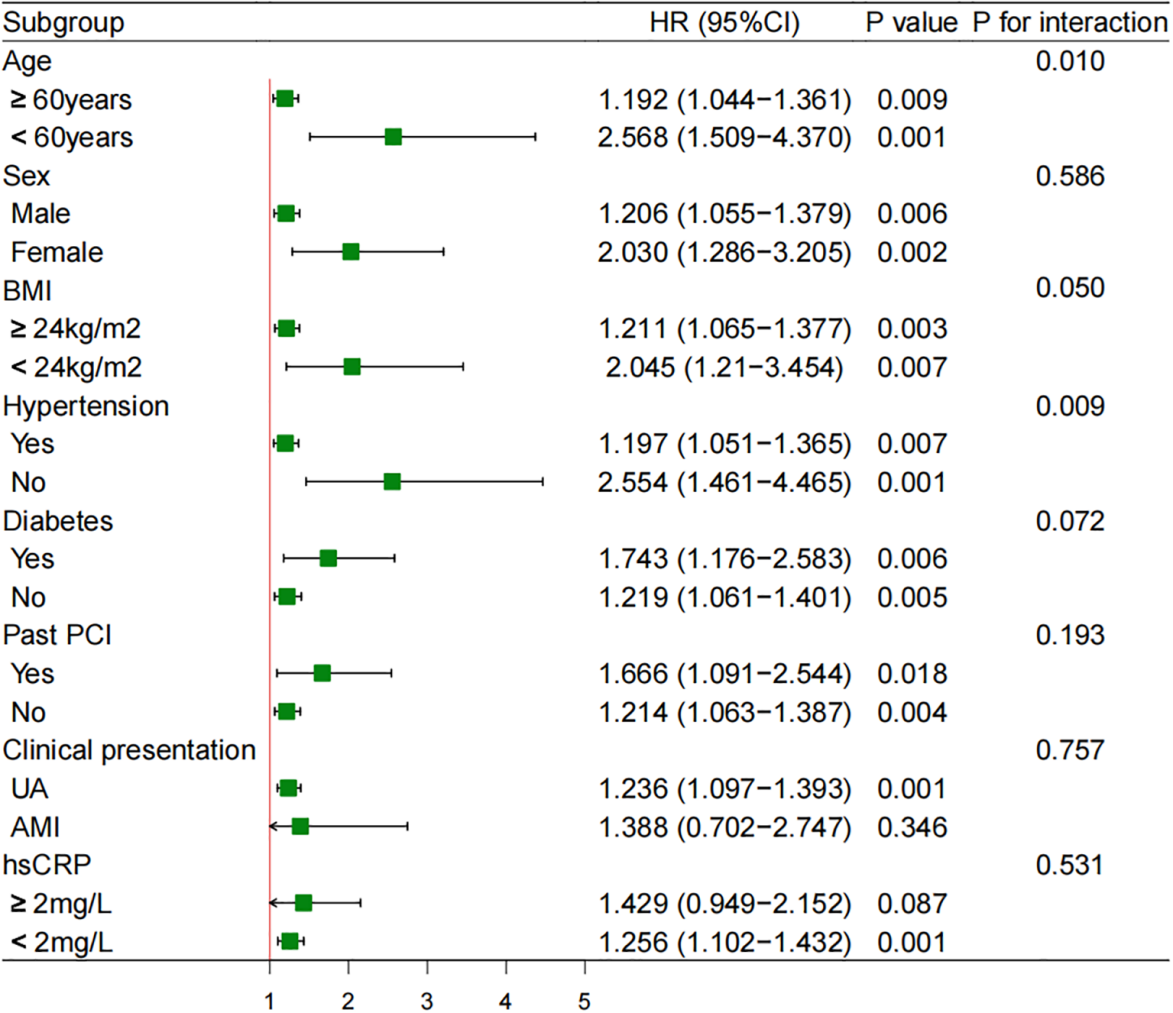
Subgroup analyses of SHR as a continuous variable for MACCE. HR was evaluated by per 1-unit increase in the SHR. AMI, acute myocardial infarction; BMI, body mass index; CI, confidence interval; HR, hazard ratio; Hs-CRP, high-sensitivity C-reactive protein; PCI, percutaneous coronary intervention; UA, unstable angina.

Subgroup analyses were performed to examine the association between SHR (as a continuous variable) and MACCE across various patient strata (**Figure 2**). The deleterious effect of a higher SHR was consistently observed in most subgroups, including those stratified by sex (male vs. female), BMI (≥ vs. < 24 kg/m^2^), diabetes status (yes vs. no), clinical presentation (UA vs. acute MI [AMI]), and hs-CRP levels (≥ vs. < 2 mg/L). Significant interactions were detected for age (≥ vs. < 60 years) and hypertension (yes vs. no), indicating that the association between SHR and MACCE was notably stronger in patients younger than 60 years (HR 2.568, 95% CI 1.509-4.370) compared to those aged 60 years or older (HR 1.192, 95% CI 1.044-1.361; *P* for interaction = 0.010), and in patients without hypertension (HR 2.554, 95% CI 1.461-4.465) compared to those with hypertension (HR 1.197, 95% CI 1.051-1.365; *P* for interaction = 0.009).

## Discussion

The present study yields several pivotal findings regarding the prognostic role of the SHR in a complex, high-risk patient cohort. We demonstrated for the first time that in patients with prior CABG who presented with an ACS and were treated with PCI, an elevated SHR was a powerful and independent predictor of long-term adverse cardiovascular outcomes. The association exhibited a clear, dose-response relationship, with patients in the highest SHR tertile facing a near two-fold increased risk of MACCE compared to those in the lowest tertile. This risk persisted after rigorous multivariate adjustment for a comprehensive panel of potential confounding factors, including GRACE risk score, several well-established cardiovascular risk factors, lipid profile, inflammatory markers, and procedural complexity. It is important to note that the observed association between SHR and MACCE appeared to be primarily driven by significant increases in all-cause death, non-fatal stroke, and unplanned revascularization. We determined the optimal cutoff value of SHR in predicting MACCE. Compared with those with SHR < 0.7889, patients with SHR ≥ 0.7889 had a markedly higher risk of developing MACCE and should receive intensive medical therapy at follow-up to reduce the risk of MACCE. Furthermore, the addition of SHR to the baseline reference prediction model including GRACE risk score significantly improved the prediction performance. Of note, compared with AFBG and HbA1c, SHR appeared to have a stronger predictive ability for MACCE. The prognostic utility of SHR was remarkably consistent across most clinically relevant subgroups, though it was notably more pronounced in younger patients and those without hypertension. This study thereby fills a critical knowledge gap by identifying SHR as a novel, robust, and readily available biomarker for risk stratification in ACS patients with prior CABG undergoing PCI.

ACS often occurs in patients with prior CABG. Compared with ACS patients without prior CABG, those with ACS and prior CABG have been shown to have more comorbidities (6); as our study shows, more than half of the study patients had diabetes, nearly 80% had hypertension, and nearly half had a history of MI. Numerous studies have shown that ACS patients with prior CABG have significantly increased long-term adverse cardiovascular outcomes compared with those without prior CABG (6). Prior observational study evidence supports an early invasive strategy for ACS patients with prior CABG (6, 30), along with the TACTICS-TIMI 18 trial reporting a reduction in MI with a routine invasive approach (31), prompting current ACS guidelines to make some positive recommendations for invasive strategy and coronary revascularization (7, 8). When patients with prior CABG require repeat coronary revascularization, PCI is more commonly used for coronary revascularization compared to repeat CABG (9). Of note, patients with prior CABG showed significant differences in the procedural characteristics and clinical outcomes associated with PCI compared to those without prior CABG, owing to altered coronary anatomy and differences in conduit pathophysiology (5). Consequently, patients with prior CABG presenting with an ACS constitute a subgroup at high cardiovascular risk and have a poor prognosis even after PCI. Our findings extend the prognostic value of SHR to the highly specific and pathophysiologically distinct subgroup, which could help to identify high-risk individuals and give aggressive secondary preventive treatment, thereby reducing the risk of adverse cardiovascular events.

The study of Xie Y et al. enrolling 2,412 patients undergoing CABG showed that SHR was an independent risk factor for the occurrence of the composite endpoint including cardiac death, nonfatal MI, nonfatal stroke, revascularization, and re-hospitalization for angina or heart failure (25). Similarly, another large-scale cohort study including 18,307 CABG patients by Li Z et al. showed that the risk of long-term MACCE increased monotonically with the increase in SHR, with no significant nonlinear relationship identified in the study population, regardless of the glycemic status (26). A report by Meng W and colleagues revealed that among 3,212 AMI patients who underwent PCI, SHR was an independent predictor of major adverse cardiovascular events, which included all-cause death, recurrent MI, target vessel revascularization, readmission for heart failure, and stroke; unfortunately, patients with prior CABG accounted for less than 2% of the total (27). All these studies have confirmed the prognostic value of SHR in patients with ACS undergoing PCI or those undergoing CABG. However, our study is the first to date to explore the prognostic value of SHR in ACS patients with prior CABG who underwent PCI.

The clinical course of patients with prior CABG is often complicated by a persistent pro-inflammatory state, diffuse endothelial dysfunction, and accelerated atherosclerosis in both native vessels and bypass grafts, particularly SVGs (3, 6, 32, 33). It has been shown that the rates of graft patency are significantly lower in stented coronary arteries compared with those without stent implantation, which can be explained in the following ways: Firstly, there may be distal microembolization from the failed stent; Secondly, the chronic inflammatory response induced by the stent may lead to downstream endothelial dysfunction and intimal hyperplasia (23). These unique milieus create a “vulnerable substrate” wherein the deleterious effects of acute metabolic stress are likely amplified. The pathophysiological mechanisms linking stress hyperglycemia to adverse outcomes—including exacerbated oxidative stress, enhanced platelet aggregation, impaired fibrinolytic capacity, and profound inflammatory activation (34, 35)—may be particularly damaging in patients whose cardiovascular system is already burdened by the sequelae of prior cardiac surgery and graft disease. SHR may be associated with accelerated atherosclerosis through the above mechanisms. The study of Liu S and colleagues enrolling 1,234 patients diagnosed with ACS who underwent stent implantation and follow-up evaluations by coronary angiography showed that SHR was closely related to the progression of non-target coronary lesions (36). Furthermore, the acute glycemic surge, as captured by a high SHR, may act as a “final trigger” that potentiates plaque instability, microvascular obstruction, and distal embolization during PCI, leading to larger infarct sizes and worse prognosis (37). The stronger association observed in younger and non-hypertensive patients is intriguing; it may suggest that in the absence of the dominant risk factors of advanced age and long-standing hypertension, the unmasked effect of acute metabolic stress becomes a primary driver of risk, highlighting the role of SHR in identifying vulnerability that traditional risk factors might miss.

The baseline characteristics provide further context for our findings. The “U-shaped” distribution of diabetes prevalence across SHR tertiles underscores that SHR captures a different dimension of risk than chronic glycemia alone. The significantly higher rate of graft PCI (especially PCI of SVGs) in the highest SHR tertile is a critical observation. It has been shown that SVG interventions are notoriously associated with higher rates of no-reflow, periprocedural myocardial infarction, and late graft failure (38). The study of Demir ÖF et al. including 223 ACS patients with prior CABG undergoing SVG-PCI showed that SHR was an independent predictor of no-reflow phenomenon development (39). This indicates that patients with high SHR not only harbor a heightened systemic metabolic and inflammatory burden but are also more likely to have degenerated graft conduits, creating a synergistic “double-hit” scenario that predisposes them to poor outcomes.

A central strength of the SHR is its ability to distinguish acute dysmetabolism from chronic glycemia (16). Our data robustly show that SHR provides incremental prognostic information beyond HbA1c and a history of diabetes. This is of paramount clinical importance. At the time of ACS presentation, identifying the patient experiencing a severe stress response, irrespective of their diabetic status, allows for a more nuanced risk assessment. This aligns with the concept that the stress hyperglycemia is a marker of physiological distress and sympatho-adrenal activation after ACS, which may be more directly cardiotoxic than background hyperglycemia (40–42).

### Clinical implications

The SHR is a simple, cost-effective, and instantly calculable parameter. Its integration into the initial clinical evaluation of ACS patients with prior CABG could substantially refine early risk stratification. Identifying the highest-risk individuals using SHR could justify several management intensifications, such as: (1) preferential use of potent P2Y12 inhibitors or other antiplatelet strategies in selected cases; (2) earlier and more comprehensive screening for microvascular dysfunction; and (3) closer long-term follow-up for the detection of recurrent ischemia.

### Future perspectives

Future research should be directed towards several key questions. First, prospective multi-center studies are needed to validate our findings and potentially develop a dedicated risk prediction model incorporating SHR for this specific population. Second, mechanistic studies exploring the pathophysiological links between SHR, endothelial dysfunction, and accelerated atherosclerosis are warranted. Third, future studies should investigate whether patients with elevated SHR benefit from stricter LDL-C targets or earlier initiation of combination lipid-lowering therapy. Of note, nearly all patients in our cohort (99.1%) were discharged on moderate-dose statin therapy. However, the persistent high event rate in patients with elevated SHR suggests that residual metabolic and inflammatory risk may not be fully addressed by standard lipid-lowering regimens. Recent guidelines support the use of more intensive lipid-lowering strategies, including high-dose statins plus ezetimibe and/or proprotein convertase subtilisin/kexin type 9 (PCSK9) inhibitors, particularly in high-risk subgroups of ACS patients (43, 44). Therefore, whether SHR can identify high-risk individuals who are able to benefit from more intensive lipid-lowering strategies warrants further exploration. Finally, and perhaps most importantly, intervention studies are warranted to investigate whether a treatment strategy targeting stress hyperglycemia—guided by the SHR—can improve outcomes. This could involve protocols for smoother and more controlled in-hospital glucose management or exploring the potential benefits of novel anti-diabetic agents with proven cardiovascular benefits (e.g., sodium-glucose cotransporter 2 [SGLT2] inhibitors, glucagon-like peptide-1 [GLP-1] receptor agonists) initiated early in the post-ACS period in these high-risk patients, as recommended in recent guidelines (43, 44).

### Study limitations

The interpretations of our study must be considered in the context of its limitations. The retrospective, single-center design, while allowing for a detailed analysis of a specific cohort, inherently carries the risk of residual confounding despite extensive statistical adjustments (45). The calculation of SHR relied on a single baseline AFBG and HbA1c measurement, which may not fully represent the dynamic nature of glycemic control or glycemic variability during the hospitalization, a factor itself linked to outcomes (46). Details on in-hospital glycemic management (e.g., insulin protocols) and long-term glycemic control after discharge were not available and could represent unmeasured confounders. Finally, as with any single-center study, the generalizability of our findings requires confirmation in larger, prospective, multi-ethnic cohorts.

## Conclusions

Among ACS patients with prior CABG undergoing PCI, an elevated SHR is a strong and independent predictor of long-term MACCE. Of note, the observed association between SHR and MACCE seemed to be mainly driven by significant increases in all-cause death, non-fatal stroke, and unplanned revascularization. Our study showed that the SHR value of 0.7889 was the critical threshold for poor prognosis. The SHR provides substantial prognostic information beyond established risk scores and traditional markers of glycemic status, underscoring the critical interplay between acute metabolic stress and cardiovascular outcomes in this complex cohort. The implementation of this readily available biomarker into clinical practice holds significant promise for improving risk stratification and paving the way for more personalized, and potentially more effective, management strategies for these high-risk individuals.

## Data Availability

The data analyzed in this study is subject to the following licenses/restrictions: The datasets used during the current study are available from the corresponding author on reasonable request. Requests to access these datasets should be directed to Zhijian Wang, zjwang1975@hotmail.com.

## Glossary

ACEI/ARB/ARNIs: angiotensin-converting enzyme inhibitors/angiotensin receptor blockers/angiotensin receptor-neprilysin inhibitors
ACS: acute coronary syndrome
AFBG: admission fasting blood glucose
BMI: body mass index
CABG: coronary artery bypass grafting
CAD: coronary artery disease
CI: confidence interval
CKD-EPI: Chronic Kidney Disease Epidemiology Collaboration
cNRI: continuous net reclassification improvement
eGFR: estimated glomerular filtration rate
FBG: fasting blood glucose
GLP-1: glucagon-like peptide-1
GRACE: Global Registry of Acute Coronary Events
HbA1c: glycosylated hemoglobin A1c
HDL-C: high-density lipoprotein cholesterol
HR: hazard ratio
Hs-CRP: high-sensitivity C-reactive protein
IDI: integrated discrimination improvement
IQR: interquartile range
LDL-C: low-density lipoprotein cholesterol
LVEF: left ventricular ejection fraction
MACCE: major adverse cardiovascular and cerebrovascular events
MI: myocardial infarction
NSTEMI: non-ST-segment elevation myocardial infarction
PAD: peripheral artery disease
PCI: percutaneous coronary intervention
PCSK9: proprotein convertase subtilisin/kexin type 9
ROC: receiver operating characteristic
SBP: systolic blood pressure
SCr: serum creatinine
SD: standard deviation
SGLT2: sodium-glucose cotransporter 2
SHR: stress hyperglycemia ratio
STEMI: ST-segment elevation myocardial infarction
SVG: saphenous vein graft
TC: total cholesterol
UA: unstable angina.
