# Supplementary material for: Prognostic significance of stress hyperglycemia ratio in acute coronary syndrome patients with prior coronary artery bypass grafting
Source: Front Endocrinol (Lausanne). 2026 Jan 16;16:1741291. doi: 10.3389/fendo.2025.1741291 (PMC12855041; doi:10.3389/fendo.2025.1741291)
Supplement: Supplementary file 1 [file Table1.docx]

**Table S1. Univariate and multivariate Cox proportional hazards models including GRACE risk score for predicting MACCE according to the SHR as continuous variable**

|  | **Univariate analysis** | | **Multivariate analysis** | |
| --- | --- | --- | --- | --- |
| **Variables** | **HR (95% CI)** | **P value** | **HR (95% CI)** | **P value** |
| SHR | 1.239 (1.103-1.392) | <0.001 | 1.276 (1.105-1.474) | 0.001 |
| GRACE risk score | 1.006 (1.002-1.011) | 0.009 | 1.004 (0.999-1.009) | 0.151 |
| BMI | 1.033 (1.000-1.068) | 0.048 | 1.024 (0.990-1.060) | 0.166 |
| Hypertension | 1.302 (1.003-1.689) | 0.047 | 1.162 (0.889-1.518) | 0.272 |
| Diabetes | 1.171(0.951-1.443) | 0.137 | 1.058 (0.853-1.312) | 0.609 |
| Renal dysfunction | 1.522 (1.103-2.101) | 0.011 | 1.262 (0.886-1.798) | 0.198 |
| Past PCI | 1.312 (1.055-1.631) | 0.014 | 1.257 (0.973-1.625) | 0.080 |
| Previous stroke | 1.322 (0.980-1.782) | 0.067 | 1.261 (0.929-1.712) | 0.137 |
| Chronic lung disease | 0.654 (0.376-1.140) | 0.134 | 0.525 (0.285-0.965) | 0.038 |
| LDL-C | 1.208 (1.090-1.338) | <0.001 | 1.208 (1.083-1.347) | 0.001 |
| HDL-C | 0.526 (0.328-0.845) | 0.008 | 0.630 (0.380-1.043) | 0.073 |
| Triglycerides | 1.066 (1.013-1.121) | 0.014 | 1.041 (0.978-1.108) | 0.212 |
| Hs-CRP | 1.022 (1.008-1.037) | 0.002 | 1.014 (0.999-1.030) | 0.074 |
| Years from CABG | 1.033 (1.010-1.057) | 0.004 | 1.019 (0.994-1.045) | 0.143 |
| The index PCI as the first PCI after CABG | 0.714 (0.516-0.989) | 0.043 | 0.898 (0.600-1.345) | 0.602 |
| PCI in native and/or graft vessels |  | 0.038 |  | 0.281 |
| PCI in only native vessels | ref |  | ref |  |
| PCI in only graft vessels | 1.434 (1.063-1.935) | 0.018 | 1.654 (0.229-11.971) | 0.618 |
| PCI in both native and graft vessels | 0.810 (0.482-1.362) | 0.427 | 1.043 (0.135-8.084) | 0.968 |
| Native vessel intervened: LM | 0.685 (0.483-0.971) | 0.034 | 0.778 (0.545-1.109) | 0.165 |
| Graft vessel intervened: SVG | 1.216 (0.928-1.592) | 0.156 | 0.729 (0.099-5.348) | 0.756 |

HR indicates hazard ratio; 95% CI, 95% confidence interval. Other abbreviations as in Tables 1 and 2.
