# Supplementary material for: Prognostic significance of stress hyperglycemia ratio in acute coronary syndrome patients with prior coronary artery bypass grafting
Source: Front Endocrinol (Lausanne). 2026 Jan 16;16:1741291. doi: 10.3389/fendo.2025.1741291 (PMC12855041; doi:10.3389/fendo.2025.1741291)
Supplement: Supplementary file 2 [file Table2.docx]

**Table S2. Univariate and multivariate Cox proportional hazards models excluding GRACE risk score and including its components for predicting MACCE according to the SHR tertiles**

|  | **Univariate analysis** | | **Multivariate analysis** | |
| --- | --- | --- | --- | --- |
| **Variables** | **HR (95% CI)** | **P value** | **HR (95% CI)** | **P value** |
| SHR |  | <0.001 |  | <0.001 |
| Lowest tertile | ref |  | ref | ref |
| Middle tertile | 1.557 (1.169-2.072) | 0.002 | 1.549 (1.160-2.068) | 0.003 |
| Highest tertile | 2.145 (1.636-2.812) | <0.001 | 1.909 (1.448-2.517) | <0.001 |
| Age | 1.012 (1.000-1.025) | 0.058 | 1.013 (0.999-1.028) | 0.066 |
| BMI | 1.033 (1.000-1.068) | 0.048 | 1.020 (0.986-1.055) | 0.260 |
| SBP at admission | 1.008 (1.002-1.014) | 0.007 | 1.006 (1.000-1.012) | 0.064 |
| HR at admission | 1.016 (1.006-1.026) | 0.001 | 1.012 (1.002-1.022) | 0.021 |
| Hypertension | 1.302 (1.003-1.689) | 0.047 | 1.096 (0.835-1.439) | 0.508 |
| Diabetes | 1.171(0.951-1.443) | 0.137 | 1.029 (0.828-1.279) | 0.797 |
| Renal dysfunction | 1.522 (1.103-2.101) | 0.011 | 1.229 (0.863-1.727) | 0.288 |
| Previous MI | 1.164 (0.948-1.429) | 0.147 | 1.065 (0.860-1.319) | 0.563 |
| Past PCI | 1.312 (1.055-1.631) | 0.014 | 1.246 (0.963-1.612) | 0.094 |
| Previous stroke | 1.322 (0.980-1.782) | 0.067 | 1.208 (0.888-1.643) | 0.228 |
| Chronic lung disease | 0.654 (0.376-1.140) | 0.134 | 0.596 (0.339-1.048) | 0.072 |
| LDL-C | 1.208 (1.090-1.338) | <0.001 | 1.194 (1.068-1.335) | 0.002 |
| HDL-C | 0.526 (0.328-0.845) | 0.008 | 0.592 (0.355-0.987) | 0.044 |
| Triglycerides | 1.066 (1.013-1.121) | 0.014 | 1.026 (0.959-1.097) | 0.462 |
| Hs-CRP | 1.022 (1.008-1.037) | 0.002 | 1.015 (1.000-1.031) | 0.049 |
| Years from CABG | 1.033 (1.010-1.057) | 0.004 | 1.016 (0.990-1.042) | 0.227 |
| The index PCI as the first PCI after CABG | 0.714 (0.516-0.989) | 0.043 | 0.890 (0.594-1.335) | 0.574 |
| PCI in native and/or graft vessels |  | 0.038 |  | 0.356 |
| PCI in only native vessels | ref |  | ref |  |
| PCI in only graft vessels | 1.434 (1.063-1.935) | 0.018 | 1.512 (0.208-11.011) | 0.683 |
| PCI in both native and graft vessels | 0.810 (0.482-1.362) | 0.427 | 0.991 (0.129-7.642) | 0.993 |
| Native vessel intervened: LM | 0.685 (0.483-0.971) | 0.034 | 0.765 (0.536-1.092) | 0.140 |
| Graft vessel intervened: SVG | 1.216 (0.928-1.592) | 0.156 | 0.762 (0.104-5.601) | 0.789 |

HR indicates hazard ratio; 95% CI, 95% confidence interval. Other abbreviations as in Tables 1 and 2.
