# Supplementary material for: Prognostic significance of stress hyperglycemia ratio in acute coronary syndrome patients with prior coronary artery bypass grafting
Source: Front Endocrinol (Lausanne). 2026 Jan 16;16:1741291. doi: 10.3389/fendo.2025.1741291 (PMC12855041; doi:10.3389/fendo.2025.1741291)
Supplement: Supplementary file 3 [file Table3.docx]

**Table S3. Univariate and multivariate Cox proportional hazards models excluding GRACE risk score and including its components for predicting MACCE according to the SHR as continuous variable**

|  | **Univariate analysis** | | **Multivariate analysis** | |
| --- | --- | --- | --- | --- |
| **Variables** | **HR (95% CI)** | **P value** | **HR (95% CI)** | **P value** |
| SHR | 1.239 (1.103-1.392) | <0.001 | 1.276 (1.104-1.474) | 0.001 |
| Age | 1.012 (1.000-1.025) | 0.058 | 1.013 (0.998-1.027) | 0.081 |
| BMI | 1.033 (1.000-1.068) | 0.048 | 1.022 (0.988-1.057) | 0.213 |
| SBP at admission | 1.008 (1.002-1.014) | 0.007 | 1.006 (1.000-1.013) | 0.058 |
| HR at admission | 1.016 (1.006-1.026) | 0.001 | 1.014 (1.004-1.024) | 0.008 |
| Hypertension | 1.302 (1.003-1.689) | 0.047 | 1.093 (0.833-1.435) | 0.521 |
| Diabetes | 1.171(0.951-1.443) | 0.137 | 1.036 (0.834-1.286) | 0.749 |
| Renal dysfunction | 1.522 (1.103-2.101) | 0.011 | 1.210 (0.845-1.732) | 0.299 |
| Previous MI | 1.164 (0.948-1.429) | 0.147 | 1.064 (0.859-1.316) | 0.572 |
| Past PCI | 1.312 (1.055-1.631) | 0.014 | 1.267 (0.979-1.638) | 0.072 |
| Previous stroke | 1.322 (0.980-1.782) | 0.067 | 1.255 (0.923-1.705) | 0.147 |
| Chronic lung disease | 0.654 (0.376-1.140) | 0.134 | 0.409 (0.265-0.905) | 0.023 |
| LDL-C | 1.208 (1.090-1.338) | <0.001 | 1.192 (1.067-1.331) | 0.002 |
| HDL-C | 0.526 (0.328-0.845) | 0.008 | 0.562 (0.335-0.945) | 0.030 |
| Triglycerides | 1.066 (1.013-1.121) | 0.014 | 1.031 (0.969-1.097) | 0.339 |
| Hs-CRP | 1.022 (1.008-1.037) | 0.002 | 1.016 (1.001-1.032) | 0.039 |
| Years from CABG | 1.033 (1.010-1.057) | 0.004 | 1.016 (0.991-1.043) | 0.211 |
| The present PCI as the first PCI after CABG | 0.714 (0.516-0.989) | 0.043 | 0.886 (0.591-1.327) | 0.556 |
| PCI in native and/or graft vessels |  | 0.038 |  | 0.303 |
| PCI in only native vessels | ref |  | ref |  |
| PCI in only graft vessels | 1.434 (1.063-1.935) | 0.018 | 1.474 (0.202-10.723) | 0.702 |
| PCI in both native and graft vessels | 0.810 (0.482-1.362) | 0.427 | 0.934 (0.120-7.261) | 0.948 |
| Native vessel intervened: LM | 0.685 (0.483-0.971) | 0.034 | 0.783 (0.548-1.117) | 0.177 |
| Graft vessel intervened: SVG | 1.216 (0.928-1.592) | 0.156 | 0.831 (0.113-6.128) | 0.856 |

HR indicates hazard ratio; 95% CI, 95% confidence interval. Other abbreviations as in Tables 1 and 2.
