# Supplementary material for: Prognostic significance of stress hyperglycemia ratio in acute coronary syndrome patients with prior coronary artery bypass grafting
Source: Front Endocrinol (Lausanne). 2026 Jan 16;16:1741291. doi: 10.3389/fendo.2025.1741291 (PMC12855041; doi:10.3389/fendo.2025.1741291)
Supplement: Supplementary file 4 [file Table4.docx]

**Table S4. Assessment of multicollinearity for all variables included in the primary model**

|  | **Collinearity Statistics** | |
| --- | --- | --- |
| **Variables** | **Tolerance** | **VIF** |
| SHR tertiles | 0.978651 | 1.021815 |
| GRACE risk score | 0.836472 | 1.195497 |
| BMI | 0.922754 | 1.083713 |
| Hypertension | 0.943638 | 1.059729 |
| Diabetes | 0.959027 | 1.042723 |
| Renal dysfunction | 0.903333 | 1.107012 |
| Past PCI | 0.774486 | 1.291178 |
| Previous stroke | 0.968677 | 1.032336 |
| Chronic lung disease | 0.983806 | 1.016461 |
| LDL-C | 0.948307 | 1.054511 |
| HDL-C | 0.872675 | 1.145902 |
| Triglycerides | 0.912096 | 1.096375 |
| Hs-CRP | 0.909465 | 1.099547 |
| Years from CABG | 0.851302 | 1.174672 |
| The index PCI as the first PCI after CABG | 0.729759 | 1.370314 |
| PCI in native and/or graft vessels | 0.144687 | 6.911471 |
| Native vessel intervened: LM | 0.979920 | 1.020491 |
| Graft vessel intervened: SVG | 0.143277 | 6.979504 |

VIF indicates variance inflation factor. Other abbreviations as in Tables 1 and 2.
